# Cellular microRNAs Repress Vesicular Stomatitis Virus but Not Theiler’s Virus Replication

**DOI:** 10.3390/v8030075

**Published:** 2016-03-10

**Authors:** Aurélie De Cock, Thomas Michiels

**Affiliations:** Université Catholique de Louvain, de Duve Institute, VIRO B1.74.07, 74 Avenue Hippocrate, B-1200 Brussels, Belgium; aurelie.decock@uclouvain.be

**Keywords:** Theiler’s virus, picornaviruses, Dicer, microRNA, evolution, interferon

## Abstract

Picornavirus’ genomic RNA is a positive-stranded RNA sequence that also serves as a template for translation and replication. Cellular microRNAs were reported to interfere to different extents with the replication of specific picornaviruses, mostly acting as inhibitors. However, owing to the high error rate of their RNA-dependent RNA-polymerases, picornavirus quasi-species are expected to evolve rapidly in order to lose any detrimental microRNA target sequence. We examined the genome of Theiler’s murine encephalomyelitis virus (TMEV) for the presence of under-represented microRNA target sequences that could have been selected against during virus evolution. However, little evidence for such sequences was found in the genome of TMEV and introduction of the most under-represented microRNA target (miR-770-3p) in TMEV did not significantly affect viral replication in cells expressing this microRNA. To test the global impact of cellular microRNAs on viral replication, we designed a strategy based on short-term *Dicer* inactivation in mouse embryonic fibroblasts. Short-term *Dicer* inactivation led to a >10-fold decrease in microRNA abundance and strongly increased replication of Vesicular stomatitis virus (VSV), which was used as a microRNA-sensitive control virus. In contrast, *Dicer* inactivation did not increase TMEV replication. In conclusion, cellular microRNAs appear to exert little influence on Theiler’s virus fitness.

## 1. Introduction

RNA interference (RNAi) is a key mechanism of post-transcriptional gene expression regulation in eukaryotic cells [1]. MicroRNAs (miRNAs) are short double stranded RNA molecules, formed from precursor transcripts carrying a hairpin structure. In the nucleus, this hairpin structure is cleaved off the precursor RNA by the Drosha endonuclease. The hairpin structure is then exported to the cytoplasm where it is processed by Dicer to generate mature miRNAs which are RNA duplexes of about 21 bp [2]. miRNAs are incorporated into the RNA-induced silencing complex (RISC) where one strand is degraded and the other strand acts as a guide to trigger translation arrest and/or degradation of target mRNAs. Targets are mostly determined by sequence complementarity of the seed sequence corresponding to the first eight nucleotides of the miRNA. According to the extent of base-pairing, target mRNA will undergo translation arrest or degradation [3]. Typically, miRNA can bind the 3′ UTR of dozens of cellular mRNAs and thereby fine-tune the expression of corresponding proteins. In mammals, miRNAs regulate various physiological processes such as cell proliferation, cell differentiation, cell signaling and apoptosis. Their dysfunction can lead to development abnormalities or to cancer [4]. 

In plants and insects, small interfering RNAs (siRNAs) play a crucial role in antiviral immunity [5,6]. Double stranded viral RNAs are recognized and processed by a Dicer-like enzyme into virus-derived siRNAs. These siRNAs are then loaded into the RISC complex and bind their viral RNA target with perfect base pairing to induce viral RNA degradation [7]. Plants also express RNA-dependent RNA polymerases that amplify virus-derived siRNAs to establish a systemic RNA silencing defense against viral infection [8]. In return, the majority of plant viruses and some insect viruses express proteins that interfere with the RNA silencing pathway [9,10,11]. Mutations in the RNAi machinery can increase the susceptibility of these organisms to viral infection [12,13]. Thus, in these eukaryotes, RNAi is a key antiviral mechanism.

As plant and insect viruses, some mammalian viruses have been shown to express proteins that inhibit the miRNA synthesis machinery (e.g., Influenza A virus NS1, Ebola virus VP35, Human immunodeficiency virus-1 Tat) [14,15,16,17]. These viral proteins act by binding double-stranded (ds) RNAs and thereby also inhibit other antiviral pathways that depend on dsRNA recognition, notably the interferon (IFN) pathway, which is critical to control viral infections. Thus, the biological significance of the RNAi suppressor activity of these proteins remains unclear.

It is still debated how much miRNAs can directly target viral genomes and have antiviral activity in mammals. *In vivo*, *Dicer*-deficient mice display increased susceptibility to Vesicular stomatitis virus (VSV) infection [18] due to impaired expression of miR-93 and miR-24 which directly target viral RNA. Similarly, miR-29 was reported to bind the 3′UTR of HIV mRNA, thereby inhibiting its translation and inducing its relocation into processing bodies (P bodies). These data support a direct role for host miRNAs in antiviral immunity.

In mammals, a link does exist between the miRNA and IFN pathways. Interferon beta (IFN-β) treatment of hepatocytes raises the expression of several cellular miRNAs whose seed sequence is complementary to Hepatitis C virus (HCV) RNA [19]. These miRNAs can directly target viral RNA to attenuate HCV replication. Mahajan *et al.* [20] suggest that HCV could, however, take advantage of this replication reduction to establish a persistent infection. Recently, it has been described that miR-142-3p inhibits the replication of Eastern equine encephalitis virus in murine macrophages and dendritic cells [21]. By limiting infection of hematopoietic cells, this RNA virus might limit the induction of innate immunity and thereby promote infection.

Infection of *Dicer*-deficient mouse embryonic fibroblasts (MEFs) with a large variety of viruses (e.g., Influenza A virus, VSV, HIV, Human herpes simplex virus 1) suggested that absence of cellular miRNAs had little if any impact on the replication of these viruses [22]. Bogerd *et al.* [23] concluded that human viruses evolved to escape targeting by cellular miRNAs. A recent study compared the antiviral function of miRNAs and IFN by using recombinant VSV viruses expressing either a viral suppressor of the RNAi machinery or a viral inhibitor of the IFN pathway. Inhibition of RISC-associated small RNAs resulted in a slight decrease of viral infection, likely due to a reduced repression of interferon-stimulated genes by miRNAs. In contrast, IFN inhibition strongly enhanced viral replication [23]. 

Theiler’s murine encephalomyelitis virus (TMEV or Theiler’s virus) belongs to the Picornaviridae family that includes, among others, the prototypic poliovirus. Typical picornavirus genomes are about 8000 nucleotide-long positive-stranded RNA molecules encoding a unique polyprotein. In the picornavirus cycle, replication and translation are inter-dependent as the full-length genome is used as a template for both processes. As replication occurs in semi-confined compartments known as replication complexes, it is unclear how much the viral genome would be accessible to miRNAs activity. Several studies have examined the interaction between host miRNAs and picornaviruses. Introduction of artificial miRNA target sequences in the genome of poliovirus or Coxsackie virus A21 led to a dramatic reduction of viral load *in vitro* and *in vivo*, in cells that expressed the corresponding miRNA [24,25]. *In vivo*, however, virus escape mutants rapidly emerged that lost their miRNA target sequence, suggesting that picornavirus genomes can easily adapt to minimize the impact of RNAi. On the other hand, certain picornaviruses like Foot-and-mouth disease virus (FMDV), Encephalomyocarditis virus (EMCV), Enterovirus 71 (EV71) and Coxsackie virus B3 (CVB3), naturally carry cellular miRNA binding sites in their genome [26,27,28,29]. Targeting of these sequences by cellular miRNAs generally decreases viral replication, suggesting that viruses carrying such target sequences evolved to avoid replicating in specific cell types that express high amounts of these miRNAs. In the case of CVB3, however, a miRNA targeting the viral RNA-dependent RNA polymerase coding sequence was reported to enhance virus biogenesis [30].

The aim of this study was to assess the global influence of miRNAs on Theiler’s virus replication. We first analyzed the frequency of miRNA sequences in the virus genome to test if selection against specific miRNA targets occurred during evolution. Next, we developed a transient *Dicer* inhibition system to compare viral replication levels in *Dicer*-positive and *Dicer*-negative cells.

## 2. Materials and Methods

### 2.1. Cell Culture

L929 cells (ATCC) were maintained in Dulbecco modified Eagle medium (DMEM) (Lonza Vervier, Belgium, ref 12-604F) containing 4.5 gr/L glucose, supplemented with 10% foetal calf serum (Sigma-Aldrich, Diegem, Belgium) and 100 U/mL of penicillin-streptomycin (Lonza, Vervier, Belgium).

Mouse embryonic fibroblasts (MEFs) were isolated from a *Dicer*^flox/null^ embryo derived from a *Dicer*^flox/flox^ female mouse that had been crossed with a *Dicer*^wt/null^ male (kind gift of Frédéric Lemaigre, University of Louvain, de Duve Institute, Brussels, Belgium) [31]. MEFs were isolated by standard procedures, as described previously [32] and grown in DMEM containing ultraglutamine and 4.5 gr/L glucose, supplemented with 10% of foetal calf serum and 50 units/mL of penicillin/streptomycin. Genomic DNA was then extracted from MEFs to genotype the *Dicer* gene by PCR. Briefly, cells were centrifuged for 5 min at 4 °C and then washed twice with PBS. After resuspension in 500 μL PBS, MEFs were warmed at 99 °C during 20 min followed by 10 min centrifugation. Supernatant was used for PCR amplification. The following primers were used for PCR screening: 23F-460R to amplify the floxed *Dicer* allele and 458F-460R to amplify the excised or the null *Dicer* allele (23F ATTGTTACCAGCGCTTAGAATTCC; 458F TCGGAATAGGAACTTCGTTTAAAC; 460R GTACGTCTACAATTGTCTATG). After screening, one *Dicer*^flox/null^ MEF population was immortalized by stable transfection of a pBABE-neo vector expressing the *Simian virus 40* large T antigen. 

### 2.2. Viruses

The VSV derivative expressing the green fluorescent protein (GFP) was a gift from Martin Schwemmle (University of Freiburg, Freiburg, Germany).

KJ6 and KJ7 are derivatives of the persistent DA1 strain of TMEV [33]. Both viruses carry capsid mutations that enhance L929 cell infection [34]. KJ7 additionally possesses the GFP coding sequence inserted between codons 5 and 67 of the L-coding region [32].

A KJ6 derivative (ADC43) was constructed in which 3 miR-770-3p target sequences were introduced by site-directed mutagenesis. The target site sequences thus introduced in the viral genome were those predicted for one of the randomly generated virus sequence (see below) that contained 3 miR-770-3p target sites (5′-CCGGCAGUGUCGUCCACG-3′, 5′-UUGCGCCGAACAGUCGCCCAUG-3′, 5′-CCGUGUCACGGCACGCUUACG-3′).

Viruses were propagated and titrated on BHK-21 cells [35]. Recombinant viruses used in this study are listed in Table 1.

### 2.3. miRNA Target Sequence Predictions

We compared the wild type (WT) sequence of the viral RNA with corresponding randomly generated sequences to identify target sites of miRNAs that were “under-represented” in the WT viral genome. To do this, we used the original amino acid sequence of the TMEV polyprotein that we back-translated to generate 100 different nucleotide sequences that encode the same polyprotein sequence. To avoid codon usage bias, we excluded rare codons from the computational generation of viral nucleotides sequences. Then, we used the RNA22 server to identify potential binding sites of murine miRNAs in the WT viral RNA and in the hundred back-translated nucleotide sequences [36]. 

### 2.4. Construction of a CRE Expressing Lentiviral Vector and Cell Transduction

Lentiviral vectors were derived from pCCLsin. PPT. hPGK. GFP.pre (kindly provided by Luigi Naldini, Ospedale San Raffaele—Milano, Italy) [37]. pTM945 was generated by inserting, in the backbone of this vector, a cytomegalovirus promoter, a multicloning site, an internal ribosome entry site (IRES) from TMEV and the mCherry coding sequence. The ORF of bacteriophage P1 Cre recombinase was subcloned in this plasmid using *Bsi*WI and *Xba*I restriction sites (pADC82). Lentivirus particles were generated by transient transfection of 293T cells grown in 75-cm^2^ Petri dishes as previously described [38]. Cells were transduced with lentiviral particles 4 or 8 days before infection. Lentiviral vectors used in this study are presented in Table 1.

### 2.5. Generation of L929 Cells Overexpressing miR-770-3p and Construction of a miR-142-3p Expressing Vector

A lentiviral vector was derived from pLKO.1, which drives the expression of a sequence corresponding to miR770-3p from a RNA polymerase III promoter [39]. To this end, complementary forward and reverse primers containing the short hairpin RNA (shRNA) sequence of miR-770-3p were annealed and cloned in pLKO.1, using *Age*I/*Eco*RI restriction sites. Lentiviral particles were generated, as described previously [38] and used to transduce L929 cells. RT-qPCR was used to assess the expression of miR-770-3p in transduced and mock-transduced L929 cells, using Taqman^®^ MiRNA Reverse Transcription Kit and Taqman^®^ Gene Expression Master Mix (Applied Biosystems-Thermofisher, Erembodegem, Belgium). Expression of miR-770-3p was not detected in L929 cells and was upregulated > 271 ± 68 times in the cell populations transduced with ADC48 as compared to background.

miR-142-3p was overexpressed in cells by transient transfection of pADC38, a pGIPZ (GE Dharmacon-Thermofisher, Erembodegem, Belgium) derivative that drives the expression of artificial miRNAs based on the backbone of miR30, from a RNA polymerase II promoter. To construct this plasmid, a 430 bp genomic sequence encompassing the miR-142-3p short hairpin sequence was amplified by PCR from C57BL/6 mouse genomic DNA and subcloned in pGIPZ using *Xho*I/*Bam*HI restriction sites.

### 2.6. Reporter Vectors and Luciferase Assays

As reporter vector, we used a lentiviral vector (pADC1) that expresses the *Renilla* luciferase gene derived from pGL4.78 (Promega, Leiden, The Netherlands). In the 3′ UTR region of the *Renilla* luciferase gene, we subcloned a *Bsi*WI/*Xba*I fragment carrying 4 tandem target sequences for miR-770-3p and miR-142-3p, yielding plasmids pADC30 and pADC39, respectively. Control plasmids pADC62 and pADC64 were obtained similarly by cloning 4 tandem miR-770-3p or mirR-142-3p target sequences that carry point mutations preventing their recognition by their cognate miRNA (Table 2).

As a control of transfection efficiency, we used a pcDNA3 derivative, pADC13 carrying the *Photinus* pyralis (firefly) luciferase gene from pGL2 (Promega). Transfection was performed with TransIT-LT1 reagent (Mirus Bio LLC, Madison, WI, USA) according to the manufacturer’s protocol. Luciferase assays were carried out with the Dual-Luciferase Reporter Assay System (Promega, Leiden, The Netherlands) and results are reported as relative luciferase units (Luciferase of the reporter/Luciferase of the control vector). Reporter vectors used in this study are listed in Table 1. Corresponding nucleotides sequences of miRNA binding sites are given in Table 2.

### 2.7. RNA Extraction and Quantitative RT-PCR

Total RNA preparations, reverse transcription and quantitative PCR (RT-qPCR) were performed as previously described [40]. Primers used for amplification are presented in Table 3. qPCR standards consisted of 10-fold dilutions of quantified plasmid DNA carrying the sequence to be amplified. Plasmids pTM793 (*Actb*), pCS40 (*Oasl2*), pMIP01 (*Usp18*), pSPA31 (*Stat1*) and pSPA33 (*Pkr*) correspond to pCR4-Topo (Invitrogen, Merelbeke, Belgium) derivatives carrying the corresponding amplicons. Plasmid pTM410 carries nucleotides 1–1730 of TMEV (DA1 strain), plasmid pMD2-VSV-G carries the sequence encoding VSV glycoprotein and plasmid pCDNA3-IFN-β carries the mouse gene encoding IFN-β. Reference plasmids are given in Table 3. 

Quantitative RT-PCR for miR-let-7b was performed as previously described [41,42]. Forward (ACACTCCAGCTGGGTGAGGTAGTAGGTTGT) and reverse primers (CTCAACTGGTGTCGTGGAGTCGGCAATTCAGTTGAGAACCACAC) were kindly provided by Frédéric Lemaigre (University of Louvain, de Duve Institute, Brussels, Belgium).

Expression of miR-770-3p was quantified by RT-qPCR in different tissues of C57BL/6 mice, using 10 ng of total RNA prepared previously [32]. RT was performed using TaqMan^®^ MiRNA Reverse Transcription Kit (Applied Biosystems-Thermofisher, Erembodegem, Belgium) according to the manufacturer’s protocol. 1 μL of cDNA was used to perform qPCR using the TaqMan^®^ Gene Expression Master Mix (Applied Biosystems-Thermofisher, Erembodegem, Belgium) in an ABI 7300 thermal cycler, according to the manufacturer’s protocol. PCR programs were 95 °C for 10 min, 40 cycles at 95 °C for 10 s and 60 °C for 1 min.

### 2.8. Flow Cytometry

Cells were trypsinized and resuspended in phosphate-buffered saline containing 5% of filtered fetal calf serum and 0.5% of paraformaldehyde. Data acquisition was performed on a LSRFortessa cell analyzer (BD biosciences, Erembodegem, Belgium) using the FACSDiva software. Data were analyzed using FlowJo 9.6.4. In cells transduced with mCherry-expressing vectors, viral replication (GFP fluorescence) was measured in the mCherry-positive-gated population. The rate of infection was defined as the percentage of GFP-positive cells. Viral replication was estimated by the median of GFP fluorescence intensity in the GFP-positive population. Infection efficiency was calculated by combining the rate of infection and viral replication (percentage of GFP-positive cells multiplied by median of GFP fluorescence of these cells in the mCherry-positive population).

### 2.9. Statistical Analysis

Statistical significance was calculated for at least 3 replicates, using the Student *t* test. (*, *p* < 0.005; **, *p* < 0.01; ***, *p* < 0.001).

## 3. Results

### 3.1. Under-Represented miRNA Target Sequences in TMEV Genome

It was proposed that viral genomes might have evolved to suppress miRNA target sequences in order to escape miRNA-mediated replication inhibition [43]. We analyzed whether TMEV evolved to suppress the presence of specific miRNA target sequences in its genome. Therefore, we compared the frequency of predicted miRNA target sequences that are present in the coding sequence of the DA1 strain of TMEV and in randomly generated nucleotide sequences that encode an identical polyprotein sequence.

The miR-770-3p target sequence was the most under-represented in TMEV genome (no occurrence in TMEV and a mean of 1.39 occurrences in the 100 randomly generated sequences) (Figure S1A). As this miRNA is mainly expressed in the brain and spinal cord (Figure S1B), we hypothesized that the virus could have evolved to suppress these target sequences, thus avoiding the restriction imposed by miR-770-3p on viral replication in the central nervous system. 

**Figure 1:**
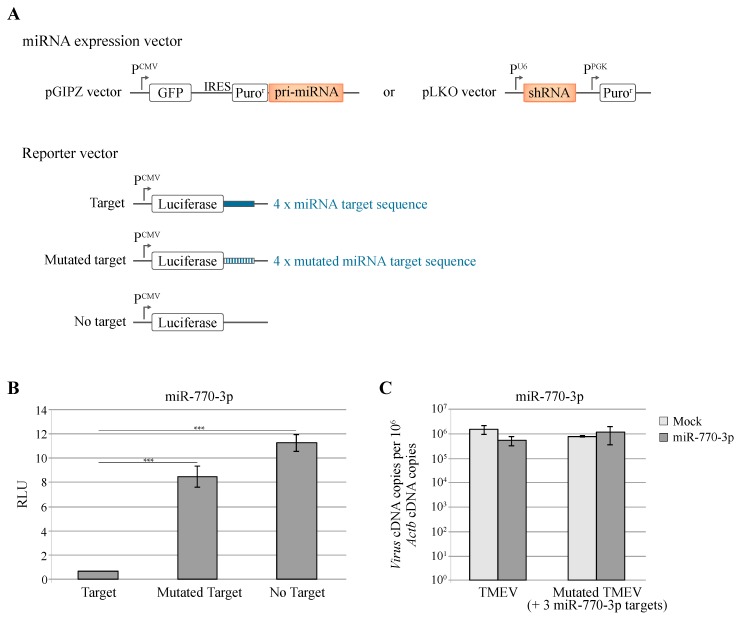
Lack of influence of miR-770-3p on TMEV replication. (**A**) Illustration of miR expression and reporter vectors. miR expression was achieved by expressing either an artificial primary miRNAs using the pGIPZ vector or a short hairpin RNA, using the pLKO.1 vector. Reporter vectors carry a *Renilla* luciferase gene with 0 (No target) or 4 wild type (Target) or mutated (Mutated target) miR target sequences; (**B**) L929 cells expressing miR-770-3p from the pLKO vector were transduced with the indicated reporter constructs and luciferase activity (Relative Luciferase Units : RLU) was assessed 24 h post transduction; (**C**) L929 cells expressing miR-770-3p were infected for 8 hours with wild type and mutant TMEV derivatives before replication analysis by RT-qPCR (**C**). Viruses were KJ6 (TMEV) and ADC43, a KJ6 derivative carrying 3 extra binding sites for miR-770-3p. Graphs show the mean and SD of a triplicate experiment (out of 2). *** *p* < 0.001

To investigate this hypothesis, we generated L929 cells that over-express miR-770-3p and constructed a TMEV mutant virus, ADC43, where 3 predicted miR-770-3p target sites were introduced by site-directed mutagenesis. Then we compared the replication of the WT and mutant viruses in L929 cells that overexpressed or not the miRNA construct (Figure 1).

shRNA expressed in these cells efficiently decreased *Renilla* luciferase expression from transfected reporter plasmids carrying the corresponding miRNA target sequences in the 3′UTR of the luciferase gene (Figure 1B).

Replication of WT and mutant viruses were assessed, by RT-qPCR, 8 h after infection. As shown in Figure 1C, the presence of miR-770-3p target sequences in the viral genome did not restrict viral replication in cells expressing this miRNA (Figure 1C). 

### 3.2. Cre-Mediated Dicer Inactivation Reduces Expression and Activity of Cellular miRNAs

**Figure 2:**
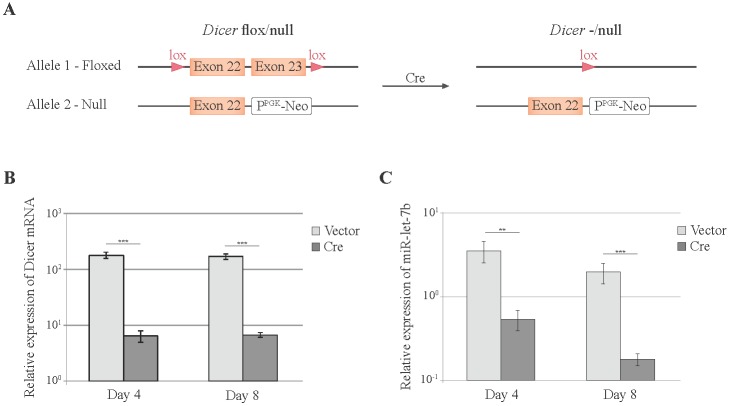
Short-term *Dicer* inactivation influence on cellular miRNA expression. (**A**) Illustration of *Dicer* alleles present in MEFs before and after Cre-mediated recombination; (**B**,**C**) Four and 8 days after transduction of *Dicer*^flox/null^ MEFs with either TM945 expressing mCherry alone (Vector) or ADC82 co-expressing mCherry and Cre (Cre), WT *Dicer* mRNA (**B**) and mature miR-let-7b (**C**) were quantified by qRT-PCR. Graphs show the mean and standard deviation (SD) of a typical experiment (out of 2) performed in triplicate. ** *p* < 0.01, *** *p* < 0.001

In order to investigate the global impact of cellular miRNAs on viral replication, we attempted to derive cells that would differ only by the expression of *Dicer*. To do so, we immortalized and cloned *Dicer*^flox/null^ MEFs, which carry one *Dicer* allele with lox sequences framing exons 22 and 23, and one *Dicer* “null” allele (*i.e.*, with a deletion of the RNaseIII domain) [31] (Figure 2A). All attempts to derive a *Dicer^−^*^/null^ cell line from these *Dicer*^flox/null^ cells, by transiently expressing the Cre recombinase from either adenovirus or plasmid vectors, led to gradual growth arrest of the cells (not shown). These cells were thus not suitable to compare viral replication in *Dicer*-positive and *Dicer*-negative cells. We therefore designed a short-term *Dicer* inactivation strategy based on Cre-expressing lentiviral vectors.

*Dicer*^flox/null^ MEFs were transduced with a lentiviral vector (ADC82) co-expressing mCherry and Cre, in order to inactivate the floxed *Dicer* allele (Figure 2A). *Dicer* expression and miRNA abundance were analyzed 4 and 8 days after transduction of the Cre vector. WT *Dicer* expression was reduced by 30-fold at both time points (Figure 2B). Abundance of the cellular miRNA miR-let-7b decreased by 7-fold at day 4 and by 11-fold at day 8 post-transduction (Figure 2C). 

**Figure 3:**
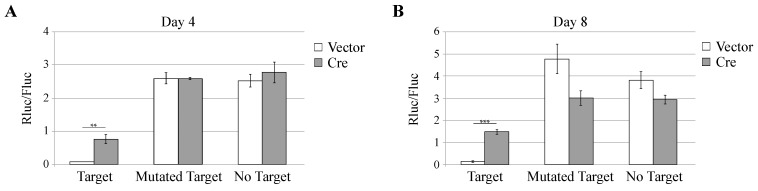
Effect of *Dicer* inactivation on miR activity. *Dicer*^flox/null^ MEFs were transduced for 4 (**A**) or 8 (**B**) days with TM945 expressing mCherry alone (Vector) or ADC82 co-expressing mCherry and Cre (Cre). Cells were then co-transfected with pADC38 (pGIPZ-miR-142-3p), with indicated *Renilla* luciferase (Rluc) reporter constructs carrying miR-142-3p targets and with pADC13 encoding firefly luciferase (Fluc) from the CMV promoter. Graphs show the mean and SD of the Rluc/Fluc ratio, which reflects the absence of miR function. ** *p* < 0.01, *** *p* < 0.001

We further tested the influence of *Dicer* inactivation on a reporter system where miR-142-3p expressed from a pGIPZ derivative inhibits the expression of a reporter luciferase construct carrying miR-142-3p target sequences. In cells transduced with the control lentiviral vector that does not express Cre, miR-142-3p triggered an important decrease of Renilla luciferase activity (Figure 3A,B). In cells transduced with the Cre-expressing vector, the decrease of luciferase activity was reduced, as expected from *Dicer* inactivation (Figure 3A,B). 

Thus, Cre expression in *Dicer*^flox/null^ MEFs induces a significant decrease of miRNAs expression and of miRNAs function that is detectable four days and more pronounced eight days after transduction.

### 3.3. Knockdown of Cellular miRNAs Increases VSV but Not TMEV Replication

**Figure 4:**
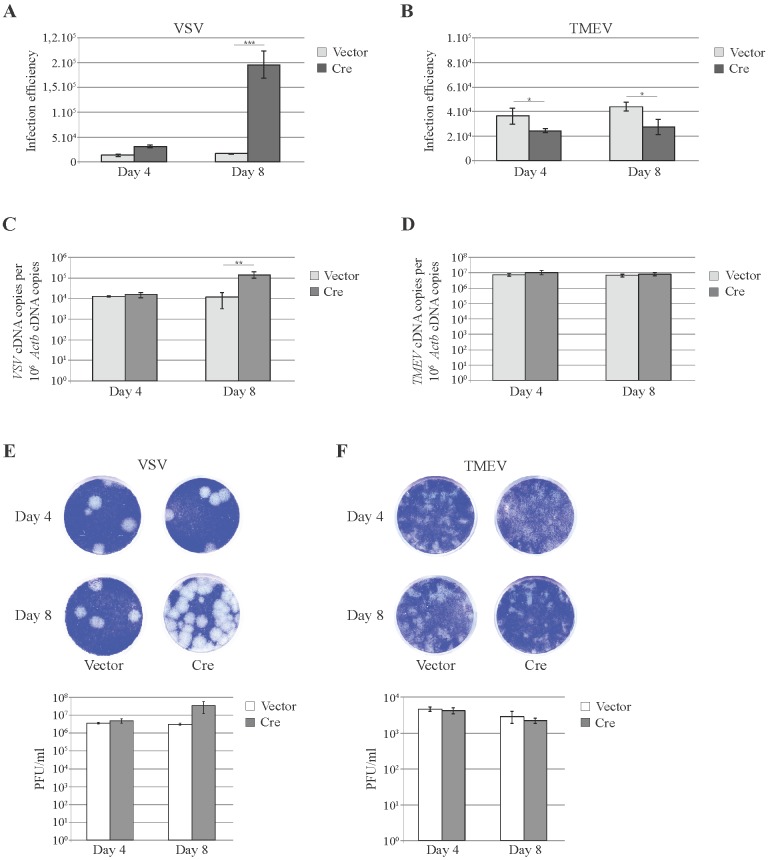
Impact of *Dicer* inactivation on VSV and TMEV infection. Infection by GFP expressing derivatives of VSV and TMEV was compared in *Dicer*^flox/null^ MEFs that were transduced for 4 or 8 days with TM945 expressing mCherry alone (Vector) or with ADC82 co-expressing mCherry and Cre (Cre). Cells were then infected in triplicate with 10 PFU per cell of VSV during 7 h or with 1 PFU per cell of TMEV during 8 h. Graphs show the mean and SD of VSV and TMEV infection efficiency as determined by flow cytometry (**A**,**B**) (1 representative experiment out of 2); RT-qPCR (**C**,**D**) or plaque assay (magnification: 0.33×) (**E**,**F**). * *p* < 0.05, ** *p* < 0.01, *** *p* < 0.001

Next, we analyzed the influence of *Dicer* inactivation on TMEV replication. VSV was used as a control since replication of this virus was reported to be susceptible to host miRNA activity. Thus, *Dicer*^flox/null^ MEFs were transduced with the lentiviral vectors co-expressing Cre and mCherry (ADC82) or expressing mCherry alone (TM945). Four and eight days after transduction, cells were infected with VSV or TMEV derivatives expressing GFP and viral replication was monitored by flow cytometry. This strategy allows the specific analysis of virus replication (GFP fluorescence) in Cre-expressing (*i.e.*, *Dicer*-/null, mCherry fluorescence) cells. As shown in Figure 4A, expression of Cre in *Dicer*^flox/null^ MEFs led to a significant increase of VSV infection efficiency at 8 days post transduction (Figure 4A). In contrast, Cre triggered a slight decrease of TMEV infection efficiency (Figure 4B). Analysis of viral replication by RT-qPCR and plaque assay confirmed that the inactivation of the floxed *Dicer* allele by CRE increased VSV but not TMEV replication (Figure 4C–F).

### 3.4. Dicer Inactivation Increases Oasl2 Expression

**Figure 5:**
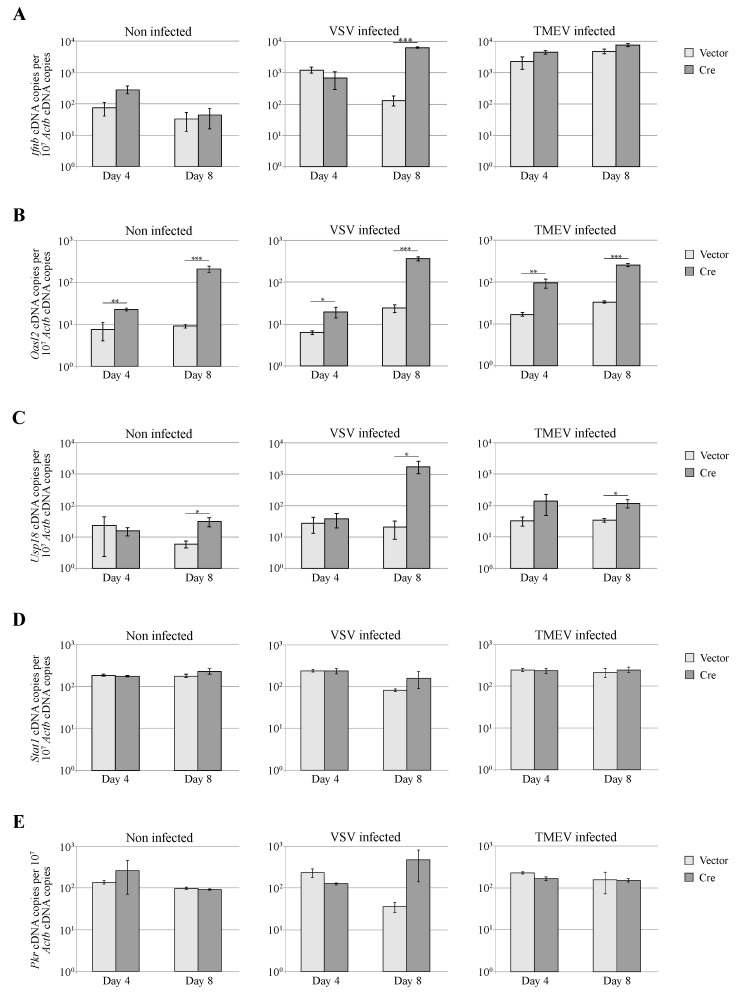
IFN response in *Dicer*-positive and *Dicer*-negative MEFs. (**A**–**E**) *Ifnb* (**A**); *Oasl2* (**B**); *Usp18* (**C**); *Stat1* (**D**) and *Pkr* (**E**) expression was quantified by RT-qPCR in non-infected, VSV-infected or TMEV-infected *Dicer*^flox/null^ MEFs transduced for 4 or 8 days with lentiviral vectors expressing mCherry alone (Vector) or co-expressing mCherry and Cre (Cre). Infection conditions were as in Figure 4.

To verify that the increase of VSV replication was not due to an impairment of the interferon response in *Dicer*-deficient cells, we used RT-qPCR to measure the expression of *Ifnb,* the gene coding interferon β and of 4 interferon-stimulated genes (ISG)(Figure 5). In non-infected cells (left graphs), *Dicer* inactivation did not influence *Ifnb*, *Stat1* or *Pkr* expression but clearly induced *Oasl2* expression. This suggests that basal *Oasl2* levels are maintained low by miRNAs, as reported by Seo *et al*. [44] who suggested that miRNAs limit ISG expression to avoid their detrimental effects on cell proliferation. As expected, infection of MEFs with either VSV or TMEV increased the expression of *Ifnb* (Figure 5A) and of *Oasl2*, *Usp18* and *Pkr* (Figure 5B,C,E). Upregulation of *Ifnb* and of ISG expression correlated with the extent of virus replication, in agreement with the fact that viral RNA is the main trigger of IFN-β expression. ISG expression was thus higher in Cre-expressing MEFs infected with VSV (Figure 5A). This shows that *Dicer* inactivation did not increase VSV replication through inhibition of the IFN response.

## 4. Discussion

In this work, we wanted to evaluate the global influence of miRNAs on TMEV replication. We first analyzed whether miRNAs shaped the viral genome sequence by selection against miRNA target sequences. The most under-represented miRNA target sequence in the TMEV genome (miR-770-3p) was absent from the viral genome and occurred at a mean frequency of 1.39 in randomly generated sequences coding the same protein, the probability to find 0 miR-770-3p target sequence in random sequences being 0.24. In addition, overexpression of a shRNA corresponding to miR-770-3p in L929 cells did not significantly affect replication of TMEV mutants where predicted miR-770-3p target sequences were introduced. The latter results must, however, be taken with caution because this shRNA was expressed from a RNA polymerase III promoter, which reportedly generates RNA that might be off one or few nucleotides [45]. The integrity of the 3′ end of the shRNA was attested by the RT-PCR procedure but alterations of the 5′ end could not be excluded. Taken together, these results fail to suggest a strong selection pressure exerted against the presence of miRNA target sequences in the genome of TMEV.

We next engineered a *Dicer* inactivation system that enables the comparison of virus replication in otherwise identical *Dicer*-positive and *Dicer*-negative cells. Complete inactivation of *Dicer* consistently led to cell growth inhibition. Therefore, short-term *Dicer* inactivation was used to analyze the impact of *Dicer* inactivation on the replication of TMEV and VSV, before the occurrence of cell growth arrest.

Eight days after Cre-mediated deletion of *Dicer*, the amount and activity of cellular miRNAs were significantly decreased. Replication of VSV was strongly enhanced while replication of TMEV was not affected. Enhancement of VSV replication in *Dicer*-negative cells was not a consequence of miRNA-mediated inhibition of the interferon antiviral response since the loss of *Dicer* did not prevent the expression of IFN-stimulated genes. Inhibition of VSV replication was thus likely a direct effect of cellular miRNAs, in line with a previous report showing that VSV can be targeted by miR93 and miR24 [18]. In contrast, TMEV replication was not increased after *Dicer* inactivation. In contrast, a very small but reproducible decrease of TMEV replication was observed. This increased TMEV replication may result from the upregulation of some ISGs, as observed for Oasl2, that may contribute to restrict viral replication.

Altogether, our results suggest that TMEV exhibits little susceptibility to the action of cellular miRNAs. A recent study showed that the IFN system was much more active than RNAi to restrict the replication of VSV [23]. In the case of TMEV, the balance between RNAi and IFN antiviral effects tips even more toward IFN, even if TMEV evolved to produce proteins that act to antagonize both IFN production and IFN effector activity [46,47]

## Figures and Tables

**Table 1:** Recombinant viruses, lentiviral vectors and reporter vectors used in this study.

Vector/Virus ^1^	Parental ^1^	Characteristics
(p)KJ6	TMEV (DA1)	Capsid adapted to infect L929 cells
(p)KJ7	(p)KJ6	GFP-coding sequence replacing codons 5–67 of L protein
(p)ADC43	(p)KJ6	TMEV carrying 3 extra target sequences for miR-770-3p
VSV-GFP	VSV	VSV carrying a GFP-coding sequence
(p)TM945	pCCLsin ^2^	Lentiviral vector carrying P_CMV_-MCS-IRES-mCherry ^2^
(p)ADC82	(p)TM945	Lentiviral vector carrying P_CMV_-Cre recombinase-IRES-mCherry ^2^
(p)ADC48	pLKO.1	Lentiviral vector expressing shRNA corresponding to miR-770-3p
(p)ADC38	pGIPZ	Lentiviral vector expressing genomic miR-142-3p
(p)ADC1	(p)TM897	Lentiviral vector carrying P_PGK_-*Renilla* luciferase ^2^
(p)ADC30	(p)ADC1	Luciferase reporter with 4 tandem miR-770-3p target sequences
(p)ADC39	(p)ADC1	Luciferase reporter with 4 tandem miR-142-3p target sequences
(p)ADC62	(p)ADC1	Luciferase reporter with 4 tandem mutated miR-770-3p target sequences
(p)ADC64	(p)ADC1	Luciferase reporter with 4 tandem copies of mutated miR-142-3p target sequences
pADC13	pcDNA3	*Photinus* luciferase (firefly) from pGL-2 basic (Promega, Leiden, The Netherlands)

^1^ names of plasmids that carry a viral genome start with a “p” while corresponding viruses lack the “p”. For example, plasmid pKJ6 carries the genome of virus KJ6; ^2^ pCCLsin = pCCLsin.PPT.hPGK.GFP.pre; PCMV = cytomegalovirus promoter; PPGK = human phosphoglycerate kinase promoter; MCS = multi-cloning site.

**Table 2:** Nucleotide sequence of miRNA binding sites incorporated in reporter vectors.

Vector	miRNA Binding Site	Nucleotide Sequence of miRNA Binding Sites
(p)ADC30	Target sequences for miR-770-3p	CCAGCTCCACGTCAGGCCCACG
(p)ADC39	Target sequence for miR-142-3p	TCCATAAAGTAGGAAACACTACA
(p)ADC62	Mutated target sequence for miR-770-3p	CCCGCACCCCGACACGCTCATG
(p)ADC64	Mutated target sequence for miR-142-3p	TCTATTAAATAAGAGACTCTTCA

**Table 3:** Primers used for gene expression analysis by RT-qPCR and reference plasmids.

Gene	Name *	Sequence 5′–3′	Plasmid
*Actb*	TM421 (Fw)	AGAGGGAAATCGTGCGTGAC	pTM793
	TM422 (Rev)	CAATAGTGATGACCTGGCCGT	
*TMEV*	TM348 (Fw)	GAACGTCAGCATTTTCCGGC	pTM410
	TM349 (Rev)	GGTGTGAAGAGCGGCAAGTG	
*VSV-G*	TM846 (Fw)	TTGCTGCTCCAATCCTCTCA	pVSV-G
	TM847 (Rev)	TCGAACACCTGAGCCTTTGA	
*Ifnb*	TM642 (Fw)	ATGAACAACAGGTGGATCCTCC	pcDNA3-IFN-β
	TM643 (Rev)	AGGAGCTCCTGACATTTCCGAA	
*Oasl2*	TM638 (Fw)	GGATGCCTGGGAGAGAATCG	pCS40
	TM639 (Rev)	TCGCCTGCTCTTCGAAACTG	
*Usp18*	TM1116 (Fw)	TGCAGGGTCTGTTCACCATC	pMIP01
	TM1117 (Rev)	GCACATGTCGGAGCTTGCTA	
*Stat1*	TM881 (Fw)	GAGGGGCCTCTCATTGTCAC	pSPA31
	TM882 (Rev)	GATTCCTGGGCTCTGTCACC	
*Pkr*	TM887 (Fw)	CGCTGGCAGAACTCAATCAC	pSPA33
	TM888 (Rev)	CTCCGGTCACGATTTGTTCA	

* (Fw) Forward and (Rev) Reverse.

## Supplementary Materials

Figure S1 is available online at www.mdpi.com/1999-4915/8/3/75/s1, Figure S1: Distribution of the number of predicted miR-770-3p targets in viral genomes and expression of miR-770-3p and miR-709 in mouse tissues.
