# Supplementary material for: Cellular microRNAs Repress Vesicular Stomatitis Virus but Not Theiler’s Virus Replication
Source: Viruses. 2016 Mar 10;8(3):75. doi: 10.3390/v8030075 (PMC4810265; doi:10.3390/v8030075)
Supplement: Supplementary file 1 [file viruses-08-00075-s001.pdf]

Supplementary Materials: Cellular microRNAs Repress Vesicular Stomatitis Virus but Not Theiler's Virus Replication

Aurélie De Cock and Thomas Michiels

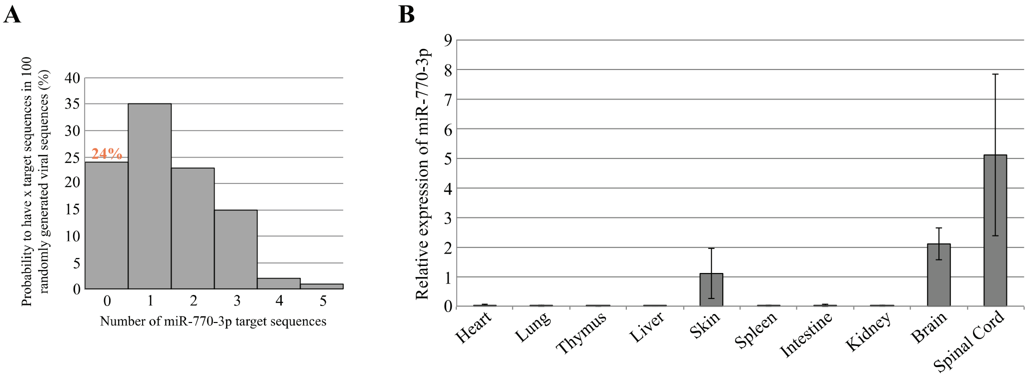

Figure S1. Distribution of the number of predicted miR-770-3p targets in viral genomes and expression of miR-770-3p in mouse tissues. (**A**) Distribution of the number of predicted miR-770-3p target sequences in 100 randomly generated sequences coding the same polyprotein. The sequence of virus DA1 contains 0 target sequence which occurs with a probability of 0.24 in randomly generated sequences; (**B**) Distribution of miR-770-3p in mouse tissues, as assessed by RT-qPCR.

© 2016 by the authors; licensee MDPI, Basel, Switzerland. This article is an open access article distributed under the terms and conditions of the Creative Commons by Attribution (CC-BY) license (http://creativecommons.org/licenses/by/4.0/).
